# Prevalence of human papillomavirus genotypes and related cervical morphological results in southern Hunan Province of China, 2018–2020: Baseline measures at a tertiary institution prior to mass human papillomavirus vaccination

**DOI:** 10.3389/fmicb.2022.1094560

**Published:** 2023-01-04

**Authors:** Zhihua Lan, Jing Zhang, Hongtao Li, Rongfang He, Qiang Zhao, Fang Yang

**Affiliations:** ^1^Department of Pathology, The First Affiliated Hospital, Hengyang Medical School of University of South China, Hengyang, China; ^2^Department of Anorectal Surgery of Traditional Chinese Medicine, The First Affiliated Hospital, University of South China, Hengyang, China

**Keywords:** human papillomavirus, genotypes, morphological examination, cervical intraepithelial neoplasm, cervical cytology, histopathology

## Abstract

**Background:**

Human papillomavirus (HPV) infection is closely correlated with cervical lesions. However, the HPV prevalence varies among different districts. This retrospective study investigated the HPV genotype distribution and its relationship with cervical lesions in southern Hunan Province.

**Methods:**

The database at our Pathology Department was searched for HPV and morphological results during 2018–2020 were reviewed. A total of 49,955 gynaecological inpatients and outpatients, each of whom underwent HPV testing based on the amplification of L1 sequence and reverse dot blot hybridization, were included in this study. Available cytology and relevant histological examination results were reviewed. Enrolled cases were categorized into seven groups based on their age. Household registry and educational level were evaluated.

**Results:**

Seven thousand two hundred eighty-six females were positive for HPV and the overall HPV positivity rate was 14.59%. The top five most prevalent HPV genotypes were HPV52, 16, 58, 53 and 51 (22.98%, 17.54%, 14.29%, 7.47%, and 5.70%, respectively). The HPV prevalence curve specific to the seven age groups showed a bimodal distribution. High school education or blow and rural residence served as risk factors for HPV infection. Single infection was the main type of HPV infection, and multiple infections occurred in 21.92% of the infected women. Of 3,148 cases had cytological results, 1,149 (36.50%) had abnormal cytological abnormalities. Among 2,833 participants with histological examination, 2000 (70.60%) had cervical abnormalities. Cytological and pathological abnormalities were mainly associated with infection with HPV16, 52 and 58. Further analysis found that HPV16, 58, 52 and 33 were the main genotypes associated with high-grade squamous lesions (HSIL) and that HPV16, 31, 33 and 58 were independent risk factors for HSIL (odd ratio [OR] = 1.70, 1.99, 2.59, 2.29; 95% CI = 1.41–2.03, 1.17–3.41, 1.88–3.59, 1.85–2.82; all *p* < 0.05). HPV16, 58, 52, 18 and 33 were the most frequent genotypes detected in squamous cell carcinoma (SCC) patients, and HPV16 and 18 were independent risk factors for cervical carcinomas (OR = 6.72;95% CI = 5.48–8.25; *p* < 0.001; OR = 1.67, 95% CI = 1.22–2.30; *p* = 0.001).

**Conclusion:**

This retrospective study demonstrated the prevalence and the distribution characteristics of HPV infection and its correlation with cervical lesions in southern Hunan Province. The comprehensive results of this survey can guide HPV vaccine research to protect against some common genotypes in China.

## Introduction

Cervical cancer, the world’s fourth most common cancer, is becoming a concerning public health problem and accounts for 40.1% of new global cases of cancer in eastern Asia (Sung et al., 2021). China, the largest developing country, reported 109,741 new cases of cervical cancer and 59,060 cervical cancer-related deaths in 2020 (Cao et al., 2021).

Human papillomavirus (HPV) is a small, nonenveloped double-stranded DNA virus. More than 200 genotypes have been identified, of which approximately 40 genotypes infect the genital tract (Bzhalava et al., 2015; Luo et al., 2020). Most HPV infections are transient and can be eradicated by the immune system. Persistent infection induces cervical lesions. Based on pathogenicity, HPVs are divided into a low-risk category, which includes HPVs that can lead to cervical low-grade squamous intraepithelial lesions (LSILs), and a high-risk category, which includes HPVs that are strong carcinogenic factors that can induce cervical high-grade squamous intraepithelial lesions (HSIL) and invasive squamous cell carcinoma (SCC). More than 90% of HSIL and SCCs are HPV-associated. Therefore, HPV vaccination has been provided to prevent cervical lesions.

Accordingly, the 2-valent vaccine against HPV16/18 and 4-valent vaccine against HPV6/11/16/18 provide 70% protection against cervical cancer. The protection rate increases to 90% in persons who receive the 9-valent vaccine targeting HPV6/11/16/18/31/33/45/52/58 (Yan et al., 2021). However, vaccine appointments are not easy, especially for the 9-valent vaccine, and there are strict age limitations in China. It is worth noting that HPV vaccines play a prophylactic but not therapeutic role in cervical epithelial lesions and that their adverse effects should not be ignored (Morimoto et al., 2015). Additionally, the geographical distributions of HPV genotypes vary from region to region. Although HPV16 is generally considered highly carcinogenic and the most prevalent genotype responsible for more than 50% of cervical cancers in most regions of the world (Bletsa et al., 2021), some eastern Asian countries, such as Korea, Japan and China, have demonstrated higher prevalence rates of HPV58 and HPV52 in women (Asato et al., 2004; Nah et al., 2017; Zhang et al., 2018).

Therefore, investigating and understanding the prevalence of HPV genotypes and their related cervical lesions in a specific region is of great importance for formulating public health strategies and guiding the application of the HPV vaccine. The first study on the prevalence of HPV in the Hengyang district demonstrated that the infection rate between April 2010 and March 2012 was 22.6% (Li et al., 2013). During 2012–2018, the overall positive rate was 18.71% (Luo et al., 2021). However, the correlation between HPV prevalence and cervical morphology in Hunan Province had not been intensively studied. The gynaecology department of the First Affiliated Hospital of University of South China, the largest medical institution in southern Hunan Province, performs HPV genotype detection in approximately 20,000 people per year. This retrospective investigation reviewed HPV detection results, the prevalence of genotypes and the characteristics of HPV-associated morphologic abnormalities to provide useful references for the detection, prevention and control of related cervical lesions in southern Hunan Province.

## Materials and methods

### Population and criteria

The database of our pathology department was searched for HPV test and related morphological examination results from HPV-positive patients from January 2018 to December 2020. We limited the interval between HPV testing and the receipt of associated morphological results (if available) in the same patient to within 180 days to ensure their correlation when analysing their relationship. The inclusion criteria were as follows: (1) history of sexual activity at any age; (2) had not been vaccinated against HPV; and (3) the first positive result from patients with several HPV test. The exclusion criteria included the following: (1) repeated results from the same patients; (2) prior physical, chemo- or radiation therapy for cervical lesions; or (3) history of conization or hysterectomy. Correlations with cytological and histological examination results after positive HPV test results were analysed.

### Cervical sample collection

Cervical samples were collected by a gynaecologist *via* two sampling brushes. The brush for the HPV test was submerged in 2 ml 0.9% saline for subsequent analysis within 24 h. The sample for the cytology test was placed into cell storage solution (Guangzhou Anbiping Medical Company Technology Co., Ltd.) for cytological testing.

### Human papillomavirus DNA genotyping

Human papillomavirus testing was carried out using the amplification of L1 sequence and reverse dot blot hybridization. HPV DNA was extracted from sampled exfoliated cervical cells and hybridized with the target HPV sequence on the hybridization chip, which included 18 high-risk HPV types (16, 18, 31, 33, 35, 45, 52, 66, 39, 58, 26, 51, 56, 59, 68, 82, 53, and 73) and 10 low-risk HPV types (6, 11, 40, 42, 44, 61, 83, 55, 43, and 81). Final result interpretation was complemented after chip washing and visualization. Chips that showed interactions with cells from the HPV-positive sample exhibited one or more blue spots at the corresponding position referring to the specific HPV genotype. The above procedures were performed with a 28 HPV GenoArray Diagnostic Kit (Guangzhou Anbiping Medical Company Technology Co., Ltd.) according to the manufacturer’s instructions.

### ThinPrep cytology test

The Sedimentation Cell Prep Plus LBC (liquid-based cytology) Processor under the liquid-based preparation (LBP) system (LBP-2601, Guangzhou Anbiping Medical Company Technology Co., Ltd.) was used. Cells from the exfoliative sample were automatically sedimented onto a glass slide, forming a diagnostic area of 13 mm in diameter. Cytology evaluation was performed in conformity with the Bethesda 2014 criteria to identify negative intraepithelial lesion or malignancy (NILM); atypical squamous cells of undetermined significance (ASC-US); atypical squamous cells, cannot exclude high-grade squamous intraepithelial lesion (ASC-H); atypical glandular cells-not otherwise specified (AGC-NOS); atypical glandular cell-favour neoplasia (AGC-FN); adenocarcinoma (AC); low-grade squamous intraepithelial lesion (LSIL); high-grade squamous intraepithelial lesions (HSIL); and SCC.

### Histological diagnosis

Cervical biopsy and other histological examination results were evaluated by two senior pathologists. Any cases with varying results were submitted to another senior doctor and confirmed by their discussion. The diagnosis was based on the 2020 World Health Organization (WHO) (Fifth Edition) classification criteria, including no neoplastic lesions, LSIL (low-grade dysplasia corresponding to cervical intraepithelial neoplasia grade I (CIN1)), HSIL (high-grade dysplasia corresponding to CIN2 and CIN3), SCC, AC (AC *in situ* and invasive AC) and adenosquamous carcinoma (ASC). If two or more pathological examination results were available for the same patient, only the most serious result was included.

### Statistical analysis

Human papillomavirus, cytological, histological and clinical data were analysed in Excel 2020 and R software (x64 4.1.2).

The HPV positivity rate, single genotype infection rate and composition of multiple genotype infections were evaluated. Analysis was performed on the basic population information, as well as household registration and educational level. The HPV-positive cases were also stratified by age group (<20, 20–29, 30–39, 40–49, 50–59, 60–69, and ≥70 years) and genotype for comparison. Correlations of different genotypes with cytological categories (NILM, ASC-US, ASC-H, LSIL, HSIL, SCC, AGC-NOS and AGC-FN) and histological categories (negative, LSIL, HSIL, SCC and AC) were calculated.

The odds ratios and relative 95% confidence intervals for each HPV genotype in HSIL and cervical cancers were calculated and compared to those of other genotypes. *p* < 0.05 was considered statistically significant.

## Results

### Gross human papillomavirus prevalence

Data from 49,955 HPV screening tests performed between 2018 and 2020 were included. 26,268 cases were from rural areas and the other 23,687 were from urban regions. The age of all the patients ranged from 14 to 91 years, with an average of 41.9 ± 11.1 years. A total of 7,286 patients, accounting for 14.59% of the total population, were HPV positive. Among them, 5,507 (75.58, 95% CI, 74.58–76.57%) were infected with high-risk genotypes, and 1779 (24.42, 95% CI, 23.43–25.42%) were infected with only low-risk genotypes. The positive rate in rural women were 16.36%, which was significantly higher than 12.62% in urban females (*p* < 0.05). Additionally, the infective rate in cases with college degree or above were 11.87%, which was significantly lower than 14.99% in females without college education (*p* < 0.05).

### Human papillomavirus prevalence by age group

**Figure 1:**
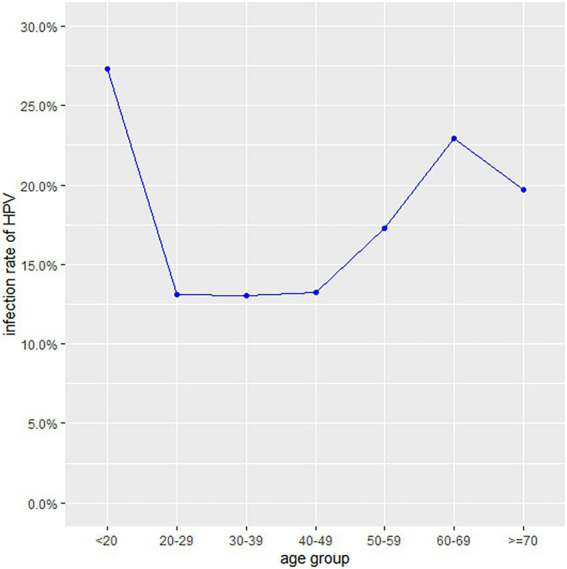
The prevalence of HPV infection in different age groups from 2018 to 2020.

The HPV prevalence curve specific to the seven age groups showed a bimodal distribution (Figure 1). The HPV infection rate of the <20 years group was 27.32% (95% CI, 23.11–31.84%) and had the highest peak. In the 20–29, 30–39 and 40–49 years age groups, the infection rates decreased were 13.14% (95% CI, 12.35–13.96%), 13.04% (95% CI, 12.48–13.61%) and 13.26% (95% CI, 12.75–13.79%), respectively. With increasing age, the prevalence rate increased to 17.25% (95% CI, 16.49–18.03%) in the 50–59 years group, with a second peak (22.93, 95% CI, 21.27–24.64%) in the 60–69 years group. The ≥70 years group accounted for 19.73% (95% CI, 16.40–23.41%) of the infected population. Regardless of age group, the positive patients were mainly infected with high-risk HPV genotypes (Table 1). A total of 22.6% (95% CI, 15.33–31.35%) of the positive patients in the <20 years group had only low-risk HPV genotypes. The rates in the 20–29, 30–39, 40–49, 50–59, 60–69 and ≥70 years groups were 15.71% (95% CI, 13.40–18.25%), 14.99% (95% CI, 13.36–16.73%), 16.09% (95% CI, 14.58–17.69%), 13.23% (95% CI, 11.61–14.98%), 9.30% (95% CI, 7.03–12.02%) and 9.71% (95% CI, 4.75–17.13%), respectively.

**Table 1:** HPV prevalence in different age groups.

Characteristics	<20, (*n*%; 95% CI)	20–29, (*n*%; 95% CI)	30–39, (*n*%; 95% CI)	40–49, (*n*%; 95% CI)	50–59, (*n*%; 95% CI)	60–69, (*n*%; 95% CI)	>70, (*n*%; 95% CI)	Total, (*n*%; 95% CI)
High-risk HPV positive	89 (77.39; 68.65–84.67)	762 (84.29; 81.75–86.60)	1,520 (85.01; 83.27–86.64)	1,852 (83.91; 82.31–85.42)	1,397 (86.77; 85.02–88.39)	507 (90.70; 87.98–92.70)	93 (90.29; 82.87–95.25)	6,220 (85.37; 84.54–86.17)
Low-risk HPV positive only	26 (22.61; 15.33–31.35)	142 (15.71; 13.40–18.25)	268 (14.99; 13.36–16.73)	355 (16.09; 14.58–17.69)	213 (13.23; 11.61–14.98)	52 (9.30; 7.03–12.02)	10 (9.71; 4.75–17.13)	1,066 (14.63; 13.83–15.46)
Total HPV positive	115 (27.32; 23.11–31.84)	904 (13.14; 12.35–13.96)	1,788 (13.04; 12.48–13.61)	2,207 (13.26; 12.75–13.79)	1,610 (17.25; 16.49–18.03)	559 (22.93; 21.27–24.64)	103 (19.73; 16.40–23.41)	7,286 (14.59; 14.28–14.90)
Negative	306 (72.68; 68.16–76.89)	5,978 (86.86; 86.04–87.65)	11,928 (86.96; 86.39–87.52)	14,436 (86.74; 86.21–87.25)	7,723 (82.75; 81.97–83.51)	1,879 (77.07; 75.35–78.72)	419 (80.27; 76.59–83.60)	42,669 (85.41; 85.10–85.72)
	421	6,882	13,716	16,643	9,333	2,438	522	49,955

**Figure 2:**
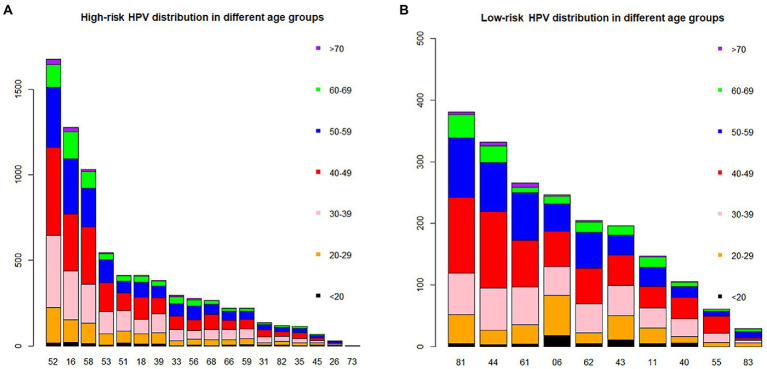
The distribution of HPV genotypes in different age groups. Each bar represents the number of infections due to corresponding HPV genotypes. Black, yellow, pink, red, blue, green and violet indicate the numbers of infections in the <20, 20–29, 30–39, 40–49, 50–59, 60–69 and >70 years groups for the corresponding genotypes, respectively. **(A)** High-risk HPV infection. **(B)** Low-risk HPV infection.

Of all the HPV genotypes, HPV52 was the most prevalent genotype found in 1674 patients, accounting for 22.98% (95% CI, 22.01, 23.96%) of infections. However, in the groups aged <20 and 60–69 years, HPV16 ranked first (Table 2; Figure 2A). Among the low-risk genotypes, HPV81 had the highest infection rate in the 40–49, 50–59, 60–69 and ≥70 years age groups. In addition, HPV06 in the groups aged <20 and 20–29 years and HPV44 in the group aged 30–39 years had the highest infection rates among the low-risk genotypes (Figure 2B).

**Table 2:** Distribution of HPV genotypes in different age groups.

HPV genotypes	<20	20–29	30–39	40–49	50–59	60–69	>=70	Positive rate in 7,286 patients (*n*%)	95% CI
High-risk HPV									
HPV16	20	134	285	332	324	155	28	1,278 (17.54)	16.67–18.43
HPV18	10	61	86	127	89	34	6	413 (5.67)	5.15–6.22
HPV26	1	4	6	8	7	5	0	31 (0.43)	1.58–2.22
HPV31	4	12	35	41	33	9	3	137 (1.88)	1.58–2.22
HPV33	3	26	66	78	75	39	10	297 (4.08)	3.63–4.56
HPV35	1	18	23	35	24	13	1	115 (1.58)	1.30–1.89
HPV39	10	68	108	94	69	30	2	381 (5.23)	4.73–5.77
HPV45	5	12	14	15	15	7	0	68 (0.93)	0.73–1.18
HPV51	18	70	118	103	70	31	5	415 (5.70)	5.17–6.25
HPV52	18	207	419	517	352	130	31	1,674 (22.98)	22.01–23.96
HPV53	6	65	128	171	133	35	6	544 (7.47)	6.87–8.09
HPV56	4	36	53	60	82	37	8	280 (3.84)	3.41–4.31
HPV58	15	120	235	337	224	97	13	1,041 (14.29)	13.49–15.11
HPV59	7	34	57	55	49	15	3	219 (3.01)	2.62–3.42
HPV66	4	32	59	55	53	16	3	222 (3.05)	2.67–3.48
HPV68	3	32	62	87	61	19	3	267 (3.66)	3.24–4.12
HPV73	0	0	1	1	0	0	0	2 (0.03)	NA
HPV82	5	23	28	24	27	7	0	114 (1.56)	1.29–1.88
Low-risk HPV									
HPV06	18	65	47	57	45	12	2	246 (3.38)	2.97–3.82
HPV11	5	25	32	35	32	16	2	147 (2.02)	1.71–2.37
HPV40	6	10	29	35	17	7	1	105 (1.44)	1.18–1.74
HPV42	5	17	47	58	58	17	3	205 (2.81)	2.45–3.22
HPV43	11	39	49	50	32	15	0	196 (2.69)	2.33–3.09
HPV44	3	24	68	124	80	27	6	332 (4.56)	4.09–5.06
HPV55	0	7	14	28	8	4	0	61 (0.84)	0.64–1.07
HPV61	4	31	61	76	78	9	7	266 (3.65)	3.23–4.11
HPV81	5	47	67	123	97	37	5	381 (5.23)	4.73–5.77
HPV83	0	6	4	4	10	4	1	29 (0.40)	0.27–0.57

### Distributions of single and multiple HPV infections in different genotype and age groups

**Figure 3:**
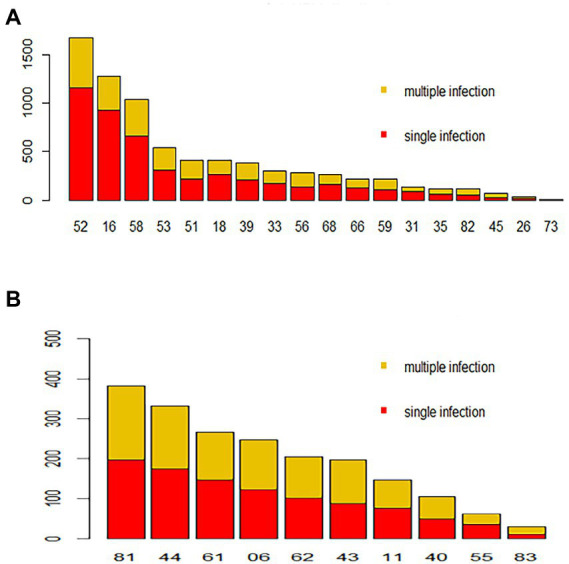
Distribution of HPV genotypes during 2018–2020. Each bar represents the number of infections due to corresponding HPV genotypes. Red indicates the number of cases with only single-type infections for the corresponding HPV genotype, and yellow indicates the number of cases due to multiple infections. **(A)** High-risk HPV infection. **(B)** Low-risk HPV infection.

**Figure 4:**
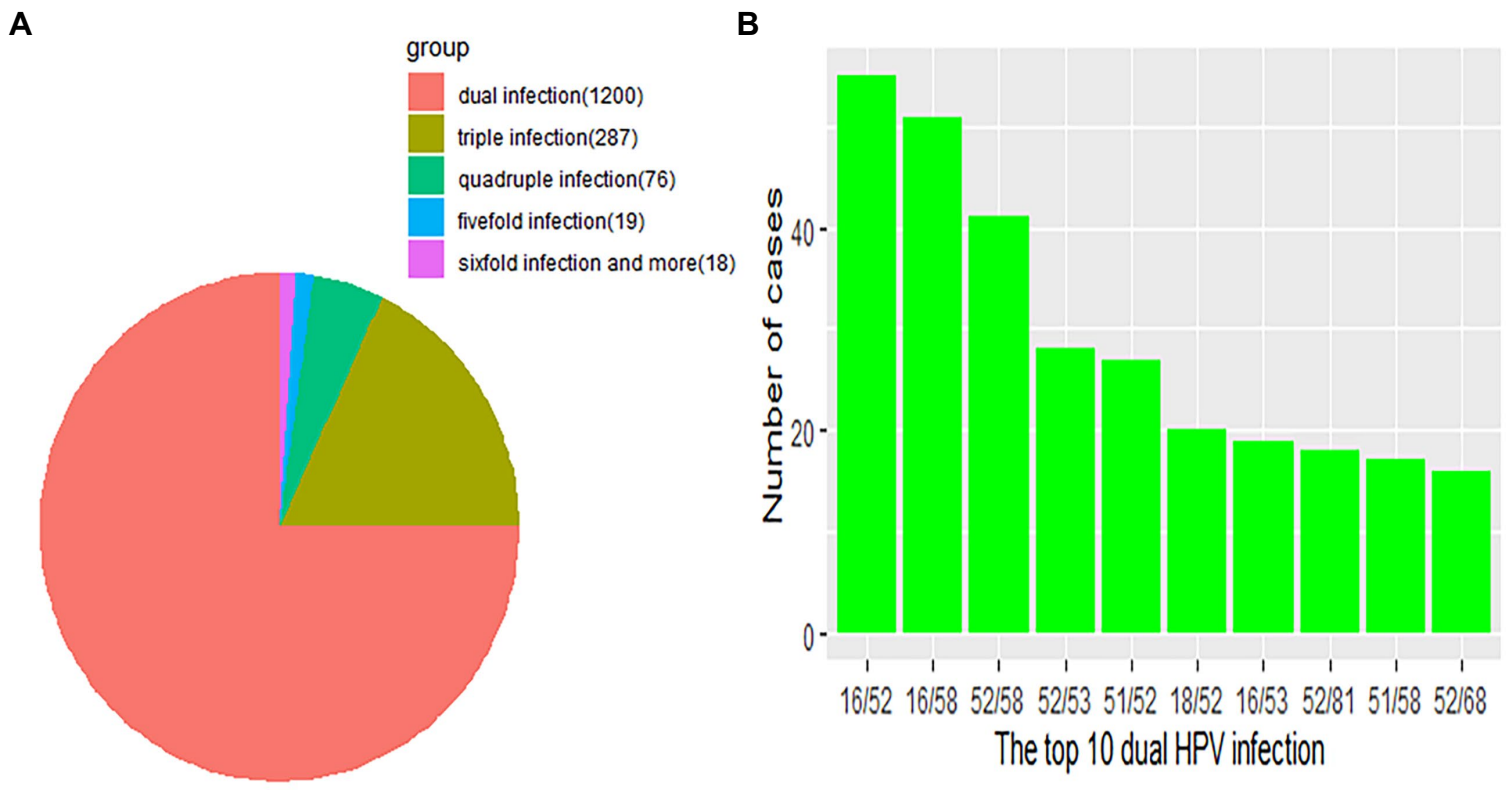
**(A)** Distribution of multitype HPV infection. Each part represents the number of cases due to multiple HPV infections. Orange, red, olivine, green, blue and violet indicate the number of dual, 3-strain, 4-strain, 5-strain, and six-strain or more infections, respectively. **(B)** The green bar represents the number of the top 10 dual HPV infections.

Regarding HPV26, 45, 56, 59, 82, 06, 40, 42 43 and 83, combinations with other genotypes were more common than single infections (Figure 3). However, single HPV infection was the most prevalent pattern in the different age groups and occurred in 5686 (78.04%; 95% CI, 77.07–78.99%) patients (Table 3). The most common multiple-infection situations were dual infections (16.50%; 95% CI, 15.62–17.34), followed by 3-strain infections (3.94%; 95% CI, 3.50–4.41%), 4-strain infections (1.04%; 95% CI, 0.82–1.30%), 5-strain infections (0.26%; 95% CI, 0.16–0.41%), and six-strain or more infections (0.25%; 95% CI, 0.15–0.39%; Figure 4A). The top 10 dual HPV combinations were as follows: 16/52, 16/58, 52/58, 52/53, 51/52, 18/52, 16/53, 52/81, 51/58 and 52/68 (Figure 4B). HPV81 was the only low-risk genotype among these 10 combinations. The multiple HPV infection rates in different age groups were different. The highest rate was in the <20 years group and reached 43.48% (95% CI, 34.26–53.04%; *p* = 0.19). As age increased, the rate decreased to 24.89% (95% CI, 22.10–27.84%), 19.02% (95% CI, 17.22–20.91%) and 18.53% (95% CI, 16.93–20.22%) in the 20–29, 30–39 and 40–49 years groups, respectively. However, the elderly groups (50–59, 60–69 and ≥70 years) accounted for 23.39% (95% CI, 21.25–25.44%), 30.77% (95% CI, 26.96–34.78%) and 28.16% (95% CI, 19.73–37.87%) of multiple HPV infections, respectively. The distribution also showed a bimodal pattern.

**Table 3:** Distribution of single-type and multiple-type infection among different age groups.

Age group	Single infection, (*n*%)	95% CI	Multiple infection, (*n*%)	95% CI
<20	65 (56.52)	(46.96–65.74)	50 (43.48)	34.26, 53.04 (*p* = 0.19)
20–29	679 (75.11)	(72.16–77.90)	225 (24.89)	22.10–27.84
30–39	1,448 (80.98)	(79.08–82.78)	340 (19.02)	17.22–20.91
40–49	1,798 (81.47)	(79.78–83.07)	409 (18.53)	16.93–20.22
50–59	1,235 (76.61)	(74.56–78.75)	375 (23.39)	21.25–25.44
60–69	387 (69.23)	(65.22–73.04)	172 (30.77)	26.96–34.78
>70	74 (71.84)	(62.13–80.27)	29 (28.16)	19.73–37.87
Total	5,686 (78.04)	(77.07–78.99)	1,600 (21.96)	21.01–22.93

### Distribution of HPV genotypes according to different cytological results

**Figure 5:**
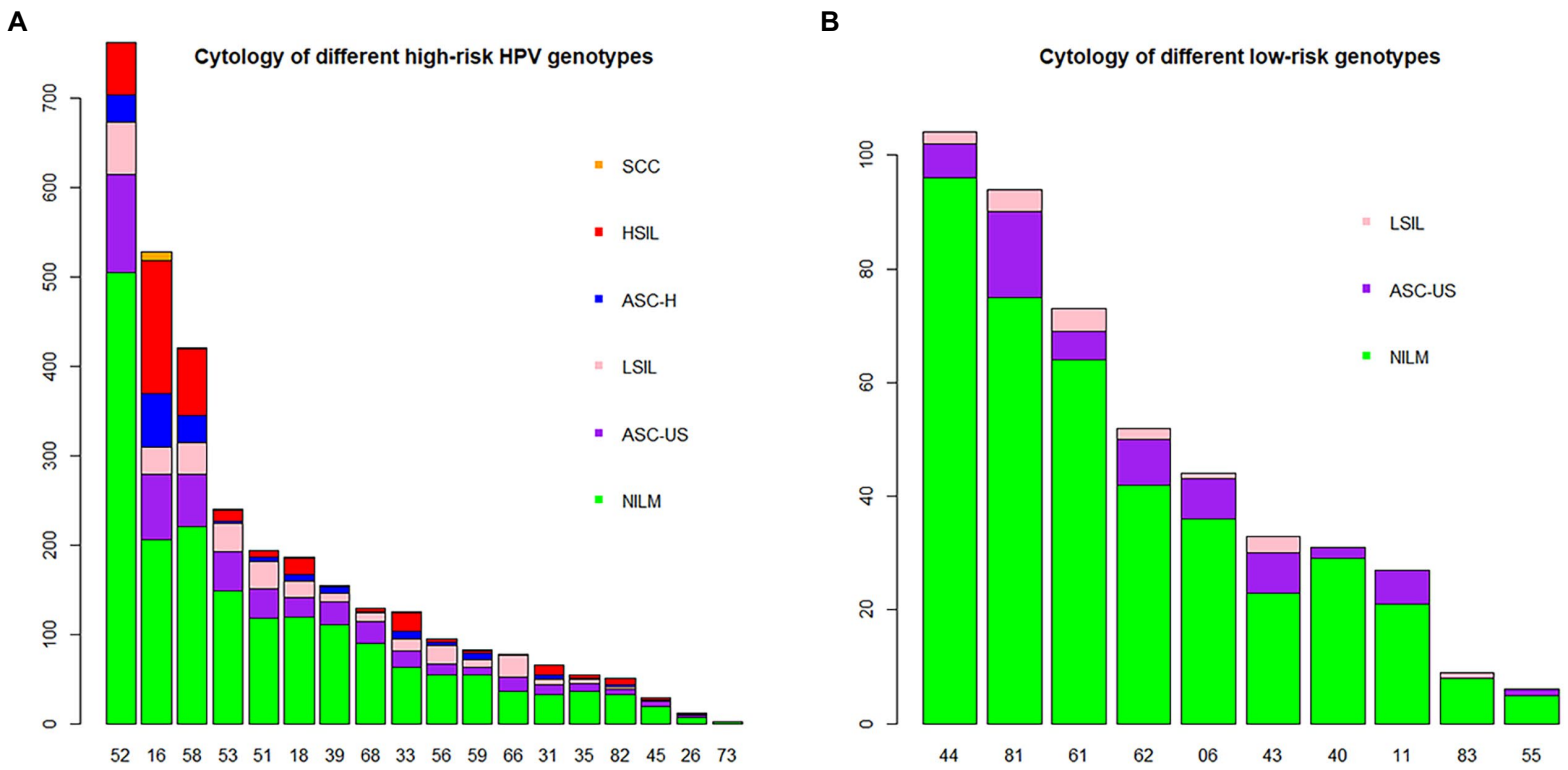
HPV genotype infection rates in the NILM, ASC-US, LSIL, ASC-H, HSIL and SCC groups. Each bar represents the number of infections due to corresponding HPV genotypes. Green, violet, pink, blue, red and yellow represent the numbers of NILM, ASC-US, LSIL, ASC-H, HSIL and SCC cases attributable to the corresponding genotype, respectively. **(A)** High-risk HPV genotypes with cytological results. **(B)** Low-risk HPV genotypes with cytological results.

To further analyse the 7,286 HPV-positive samples, cytological results from 3,148 patients in our database were analysed. The distribution of liquid-based cytological examination results of different HPV genotypes is shown in Table 4 and Figure 5. Among such patients, 1,149 (36.50%; 95% CI, 34.81–38.21%) had abnormal cytological abnormalities and 1999 (63.50%; 95% CI, 61.79–65.19%) had negative results. HPV52 was the most common genotype, accounting for 25.26% (95% CI, 23.37–27.23%) of NILM, 26.13% (95% CI, 21.99–30.60) of ASC-US and 23.32% (95% CI, 18.25–29.03) of LSIL. However, HPV16 was the leading genotype found in 44.36% (95% CI, 35.75–53.22%; *p* = 0.22) of ASC-H, 46.86% (95% CI, 41.27–50.50%; *p* = 0.29) of HSIL and 71.43% (95% CI, 41.90–90.61%; *p* = 0.18) of SCC, followed by HPV58. Among patients without high-risk HPV infection, HPV81 was the main genotype, accounting for 15 (3.54%; 95% CI, 2.01–5.81%) cases of ASC-US, accompanied by HPV61, accounting for 4 (1.58%; 95% CI, 0.43–4.00%) cases of LSIL.

**Table 4:** Distribution of HPV genotypes in different cytology results.

	NILM, (*n*%)	ASC-US, (*n*%)	LSIL, (*n*%)	ASC-H, (*n*%)	HSIL, (*n*%)	SCC, (*n*%)	AGC-NOS, (*n*%)	AGC-FN, (*n*%)	AC, (*n*%)
High-risk HPV									
HPV16	206 (10.31)	73 (17.34)	31 (12.25)	59 (44.36)	149 (46.86)	10 (71.43)	1 (33.33)	2 (40)	1 (50)
HPV18	119 (5.95)	22 (5.23)	19 (7.51)	7 (5.26)	18 (5.66)	1 (7.14)	0 (0)	2 (40)	1 (50)
HPV26	7 (0.35)	2 (0.48)	1 (0.40)	0 (0)	1 (0.31)	1 (7.14)	0 (0)	0 (0)	0 (0)
HPV31	32 (1.60)	11 (2.61)	7 (2.77)	5 (3.76)	10 (3.14)	0 (0)	0 (0)	0 (0)	0 (0)
HPV33	63 (3.15)	18 (4.28)	14 (5.53)	8 (6.02)	21 (6.60)	1 (7.14)	0 (0)	1 (20)	0 (0)
HPV35	36 (1.80)	9 (2.14)	5 (1.98)	1 (0.75)	4 (1.26)	0 (0)	0 (0)	0 (0)	0 (0)
HPV39	111 (5.55)	25 (5.94)	10 (3.95)	7 (5.26)	1 (0.31)	0 (0)	0 (0)	0 (0)	0 (0)
HPV45	19 (0.95)	6 (1.43)	1 (0.40)	0 (0)	3 (0.94)	0 (0)	0 (0)	0 (0)	0 (0)
HPV51	118 (5.90)	33 (7.84)	31 (12.25)	4 (3.01)	8 (2.52)	0 (0)	1 (33.33)	0 (0)	0 (0)
HPV52	505 (25.26)	110 (26.13)	59 (23.32)	30 (22.56)	58 (18.24)	1 (7.14)	0 (0)	0 (0)	0 (0)
HPV53	148 (7.40)	44 (10.45)	32 (12.65)	3 (2.26)	12 (3.77)	1 (7.14)	0 (0)	0 (0)	0 (0)
HPV56	54 (2.70)	13 (3.09)	21 (8.30)	3 (2.26)	4 (1.26)	0 (0)	0 (0)	0 (0)	0 (0)
HPV58	221 (11.06)	58 (13.78)	35 (13.83)	31 (23.31)	75 (23.58)	1 (7.14)	1 (33.33)	0 (0)	0 (0)
HPV59	54 (2.70)	9 (2.14)	9 (3.56)	7 (5.26)	2 (0.63)	1 (7.14)	0 (0)	0 (0)	0 (0)
HPV66	36 (1.80)	16 (3.80)	25 (9.88)	0 (0)	1 (0.31)	0 (0)	0 (0)	0 (0)	0 (0)
HPV68	90 (4.50)	24 (5.70)	10 (3.95)	1 (0.75)	4 (1.26)	0 (0)	0 (0)	0 (0)	0 (0)
HPV73	2 (0.10)	0 (0)	0 (0)	0 (0)	0 (0)	0 (0)	0 (0)	0 (0)	0 (0)
HPV82	32 (1.60)	6 (1.43)	3 (1.19)	2 (1.50)	8 (2.52)	0 (0)	0 (0)	0 (0)	0 (0)
Low-risk HPV only									
HPV06	36 (1.80)	7 (1.66)	1 (0.40)	0 (0)	0 (0)	0 (0)	0 (0)	0 (0)	0 (0)
HPV11	21 (1.05)	6 (1.43)	0 (0)	0 (0)	0 (0)	0 (0)	0 (0)	0 (0)	0 (0)
HPV40	29 (1.45)	2 (0.48)	0 (0)	0 (0)	0 (0)	0 (0)	0 (0)	0 (0)	0 (0)
HPV44	96 (4.80)	6 (1.43)	2 (0.79)	0 (0)	0 (0)	0 (0)	0 (0)	0 (0)	0 (0)
HPV42	42 (2.10)	8 (1.90)	2 (0.79)	0 (0)	0 (0)	0 (0)	0 (0)	0 (0)	0 (0)
HPV61	64 (3.20)	5 (1.19)	4 (1.58)	0 (0)	0 (0)	0 (0)	0 (0)	0 (0)	0 (0)
HPV83	8 (0.40)	0 (0)	1 (0.40)	0 (0)	0 (0)	0 (0)	0 (0)	0 (0)	0 (0)
HPV55	5 (0.25)	1 (0.24)	0 (0)	0 (0)	0 (0)	0 (0)	0 (0)	0 (0)	0 (0)
HPV43	23 (1.15)	7 (1.66)	3 (1.19)	0 (0)	0 (0)	0 (0)	0 (0)	0 (0)	0 (0)
HPV81	75 (3.75)	15 (3.56)	4 (1.58)	0 (0)	0 (0)	0 (0)	0 (0)	0 (0)	0 (0)

### Distribution of HPV genotypes according to different pathological results

**Figure 6:**
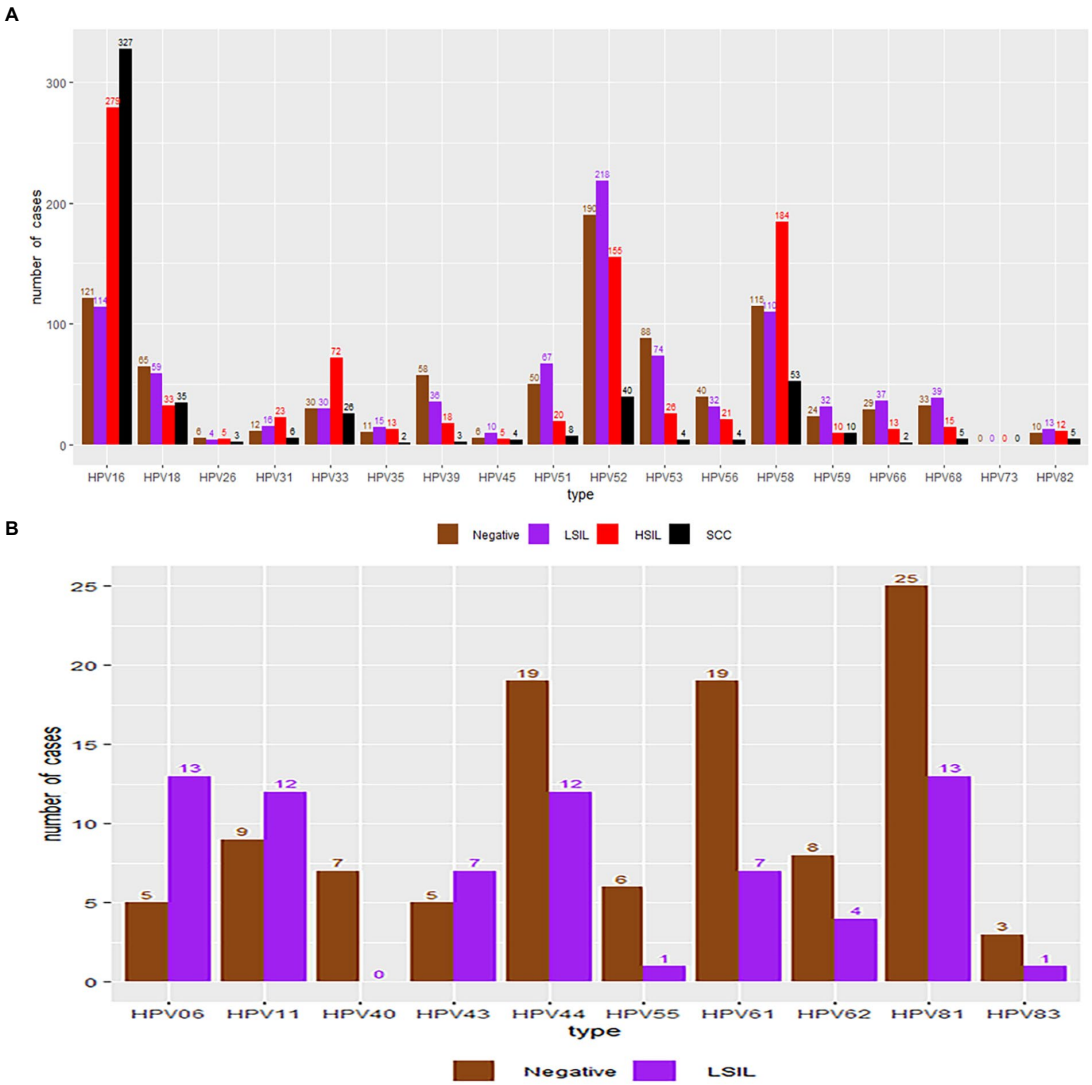
HPV genotype infection rates in the negative, LSIL, HSIL and SCC groups. Each bar represents the number of infections due to the corresponding HPV genotypes. Grey, violet, red and black represent negative results, LSIL, HSIL and SCC for the corresponding genotype, respectively. **(A)** High-risk HPV genotypes with pathological results. **(B)** Low-risk HPV genotypes with pathological results.

A total of 2,833 participants were referred for histological examination. Among them, 2,000 patients had pathological abnormalities (70.60, 95% CI, 68.88–72.27%). The distribution of pathological results of cases due to different HPV genotypes is shown in Table 5 and Figure 6. Of the 760 cases of LSIL, HPV52 was the dominant genotype (28.68%; 95% CI, 25.49–32.04%), followed by HPV16 (15.00%; 95% CI, 12.54–17.74%), HPV58 (14.47%; 95% CI, 12.05–17.18%) and HPV53 (9.74%; 95% CI, 7.72–12.07%). Of the 714 cases of HSIL, HPV16 was the dominant genotype (39.22%; 95% CI, 35.62–42.91%), followed by HPV58 (25.77%; 95% CI, 22.60–29.14%), HPV52 (21.71%; 95% CI, 18.74–24.92%) and HPV33 (10.08%; 95% CI, 7.97–12.53%). Of the 481 cases of SCC, HPV16 was the dominant genotype (67.98%; 95% CI, 63.61–72.13%), followed by HPV58 (11.02%; 95% CI, 8.36–14.16%), HPV52 (8.32%; 95% CI, 6.01–11.15%) and HPV18 (7.28%; 95% CI, 5.12–9.97%). Of the 44 cases of AC, HPV18 (50.00%; 95% CI, 34.56–65.43%; *p* = 1.00) and HPV16 (43.18%; 95% CI, 28.35–58.97; *p* = 0.45) were the main genotypes. As shown in Table 6, patients infected with HPV16, 31, 33 and 58 had increased risks of HSIL (OR = 1.70, 95% CI = 1.41–2.03; OR = 1.99, 95% CI = 1.17–3.41; OR = 2.59, 95% CI = 1.88–3.59; OR = 2.29, 95% CI = 1.85–2.82). Patients with HPV16 and 18 were at higher risk of cervical carcinoma (including SCC, AC and ASC; OR = 6.72, 95% CI = 5.48–8.25; OR = 1.67, 95% CI = 1.22–2.30).

**Table 5:** Distribution of HPV genotypes in different pathological result.

	Negative, (*n*%)	LSIL, (*n*%)	HSIL, (*n*%)	SCC, (*n*%)	AC, (*n*%)	ASC, (*n*%)
High-risk HPV						
HPV16	121 (14.53)	114 (15.00)	280 (39.22)	327 (67.98)	19 (25.00)	4 (66.67)
HPV18	65 (7.80)	59 (7.76)	33 (4.62)	35 (7.28)	22 (34.09)	1 (16.67)
HPV31	12 (1.44)	16 (2.11)	23 (3.22)	6 (1.25)	0 (0)	0 (0)
HPV33	30 (3.60)	30 (3.95)	72 (10.08)	26 (5.41)	2 (4.55)	0 (0)
HPV35	11 (1.32)	15 (1.97)	13 (1.82)	2 (0.42)	0 (0)	0 (0)
HPV45	6 (0.72)	10 (1.32)	5 (0.70)	4 (0.83)	0 (0)	0 (0)
HPV52	190 (22.81)	218 (28.68)	155 (21.71)	40 (8.32)	3 (6.82)	0 (0)
HPV66	29 (3.48)	37 (4.87)	13 (1.82)	2 (0.42)	0 (0)	0 (0)
HPV39	58 (6.96)	36 (4.74)	18 (2.52)	3 (0.62)	1 (2.27)	0 (0)
HPV58	115 (13.81)	110 (14.47)	184 (25.77)	53 (11.02)	2 (4.55)	0 (0)
HPV26	6 (0.72)	4 (0.53)	5 (0.70)	3 (0.62)	0 (0)	0 (0)
HPV51	50 (6.00)	67 (8.82)	20 (2.80)	8 (1.66)	0 (0)	0 (0)
HPV56	40 (4.80)	32 (4.21)	21 (2.94)	4 (0.83)	1 (2.27)	0 (0)
HPV59	24 (2.88)	32 (4.21)	10 (1.40)	10 (2.08)	0 (0)	1 (16.67)
HPV68	33 (3.96)	39 (5.13)	15 (2.10)	5 (1.04)	0 (0)	0 (0)
HPV82	10 (1.20)	13 (1.71)	12 (1.68)	5 (1.04)	0 (0)	1 (16.67)
HPV53	88 (10.56)	74 (9.74)	26 (3.64)	4 (0.83)	0 (0)	0 (0)
HPV73	0 (0)	0 (0)	0 (0)	0 (0)	0 (0)	0 (0)
Low-risk HPV only						
HPV06	5 (0.60)	13 (1.71)	0 (0)	0 (0)	0 (0)	0 (0)
HPV11	9 (1.08%)	12 (1.58)	0 (0)	0 (0)	0 (0)	0 (0)
HPV40	7 (0.84%)	0 (0)	0 (0)	0 (0)	0 (0)	0 (0)
HPV42	8 (0.96%)	4 (0.53)	0 (0)	0 (0)	0 (0)	0 (0)
HPV43	5 (0.60%)	7 (0.92)	0 (0)	0 (0)	0 (0)	0 (0)
HPV44	19 (2.28%)	12 (1.58)	0 (0)	0 (0)	0 (0)	0 (0)
HPV55	6 (0.72%)	1 (0.13)	0 (0)	0 (0)	0 (0)	0 (0)
HPV61	19 (2.28%)	7 (0.92)	0 (0)	0 (0)	0 (0)	0 (0)
HPV81	25 (3.00%)	13 (1.71)	0 (0)	0 (0)	0 (0)	0 (0)
HPV83	3 (0.36%)	1 (0.13)	0 (0)	0 (0)	0 (0)	0 (0)

**Table 6:** Odds ratios of high-risk HPV genotypes in HSIL and cervical cancers.

HPV genotypes	HSIL			Cervical cancers		
	Odd ratio	95% CI	*p*-value	Odd ratio	95% CI	*p*-value
HPV16	1.7	1.41–2.03	<0.001	6.72	5.48–8.25	<0.001
HPV18	0.56	0.38–0.81	0.002	1.67	1.22–2.30	0.001
HPV31	1.99	1.17–3.41	0.01	0.64	0.22–1.49	0.294
HPV33	2.59	1.88–3.59	<0.001	1.16	0.74–1.78	0.477
HPV35	1.39	0.72–2.69	0.33	0.22	0.05–0.91	0.022
HPV45	0.74	0.28–1.98	0.55	0.82	0.28–2.41	0.724
HPV52	1.04	0.85–1.28	0.703	0.27	0.19–0.38	<0.001
HPV66	0.56	0.31–1.02	0.055	0.11	0.03–0.43	<0.001
HPV39	0.53	0.32–0.89	0.015	0.15	0.05–0.40	<0.001
HPV58	2.29	1.85–2.82	<0.001	1.15	0.41–3.22	0.797
HPV26	1.15	0.41–3.22	0.797	0.87	0.25–3.00	0.821
HPV51	0.46	0.29–0.74	0.001	0.24	0.10–0.49	<0.001
HPV56	0.81	0.49–1.32	0.386	0.23	0.09–0.56	<0.001
HPV59	0.44	0.22–0.85	0.013	0.72	0.38–1.37	0.31
HPV68	0.57	0.33–1.00	0.276	0.24	0.10–0.60	0.001
HPV82	1.23	0.63–2.43	0.541	0.74	0.31–1.77	0.497
HPV53	0.45	0.29–0.68	<0.001	0.09	0.03–0.23	<0.001

## Discussion

Action toward achieving the global elimination of cervical cancer has been proposed by the World Health Organization (WHO) since 2018 (Kumar et al., 2019). Free screening for cervical cancer and breast cancer has been offered to rural women in China since 2009. At the beginning of 2022, urban women were also enrolled in the programme. As a sensitive method, HPV testing is usually used in combination with cytology for screening for cervical lesions (Hamers et al., 2022).

The fact that the HPV prevalence varies considerably on the basis of geography and population has prompted many investigations into regional epidemical strategies. Disparities also exist within countries.

The overall HPV infection rate in our study was 14.59%, which was lower than those in Guizhou (16.95), Sichuan (23.84%), Fujian (20.57%), Guangdong (19.81%), Shanghai (17.92%), Shandong (28.4%), Liaoning (16.1%), Henan (19.7%), and Jiangsu (26.92%) but higher than those in Xinjiang (14.02%) and Yunnan (12.9%; Li et al., 2016, 2020; Sun et al., 2017; Zhao et al., 2018; Chen et al., 2019; Jiang et al., 2019; Wang et al., 2019,^ 2022^; Zhang et al., 2019,^ 2020^; Luo et al., 2020). Compared to previous study, the HPV positive rate decreased significantly, which may be due to increasing health awareness led to increase participation in cervical screen (Luo et al., 2021). The distribution of the age-specific HPV infection rate presented a bimodal pattern, which was in accordance with the results of the majority of previous studies. The <20 years age group had the highest infection rate, possibly because new sexual behaviour increases the risk of virus exposure and an undeveloped immune system has an insufficient capability for virus clearance (Bergqvist et al., 2021; Yan et al., 2021). The 60–69 years age group had the second highest HPV infection rate. The reason could be a decreased ability to clear recent infection due to age-related immune senescence and persistent infections in older women from earlier exposures (Kang et al., 2014). Notably, the numbers of participants aged <20 and ≥60 were significantly less than those in the other groups. In fact, only a small minority of women under the age of 20 reported sexual behaviour. Fewer elderly women aged 60 years or older received HPV testing in the clinic, possibly because of reluctance due to an insufficient understanding or a lack of symptoms (He and He, 2020). Consistent with previous study (Yang et al., 2022), the current study shows that women with college education or from urban areas had lower risk of HPV infection. Higher education and convenient urban medical service may improve the health attention and practice of the population.

Single-type HPV infection and high-risk HPV infection in our study were the most common types of infections. The most common genotype worldwide is HPV16 (Crow, 2012), similar to that in northernmost China (Jiang et al., 2019; Zhang et al., 2020; Wang et al., 2022). However, the most prevalent genotype in southernmost China is HPV52 (Li et al., 2016, 2020; Chen et al., 2019; Zhang et al., 2019; Luo et al., 2020). In the south of Hunan province, the most common genotype has been changed from HPV 16 to HPV 52 (Li et al., 2013; Luo et al., 2021). Regardless of whether HPV16 or 52 ranks first, the top three genotypes in China are HPV16, 52 and 58 (Li et al., 2016, 2020; Sun et al., 2017; Zhang et al., 2018, 2019, 2020; Chen et al., 2019; Jiang et al., 2019; Wang et al., 2019,^ 2022^; Luo et al., 2020). As the second most carcinogenic genotype in the world, HPV18 ranks only fourth in some Chinese areas or is not even in the top five in other provinces (Li et al., 2016; Zhang et al., 2018). Moreover, HPV53 is the fourth or fifth most prevalent genotype in most areas of China (Sun et al., 2017; Chen et al., 2019,^ 2021^; Jiang et al., 2019; Wang et al., 2019,^ 2022^; Zhang et al., 2019,^ 2020^; Li et al., 2020; Luo et al., 2020), and is the most prevalent genotype among healthy women in other Asian countries (Ouh et al., 2018). Our study also demonstrated that HPV81 was the most common low-risk type, with the same overwhelming advantage in other Chinese provinces (Zhao et al., 2018; Chen et al., 2019; Zhang et al., 2019; Luo et al., 2020; Yan et al., 2021).

The multiple-infection rate in HPV-positive patients in the different age groups also showed a bimodal distribution. The peak occurred in the <20 years group, and the second highest rate was observed in the 60–69 years group, similar to the distribution of the age-specific HPV infection rate and likely explained by the same reasons (Li et al., 2020). Patients with multiple HPV infections had a longer duration of HPV infection and an increased risk of cervical lesions compared to patients with a single HPV infection (Kim et al., 2021). High-risk HPVs were also the predominant genotypes in coinfections. Among the top 10 dual infections, HPV82 was the only low-risk genotype. However, the oncogenic potential of each HPV genotype under coinfection conditions could not be assessed accurately because interactions and competition between the genotypes were unclear (Bernard et al., 2013).

Cytology screening in patients with a high-risk HPV genotype is the conventional method (Rebolj et al., 2022). The distribution of different cervical cytology results in each HPV genotype group, especially the high-risk genotypes, is helpful in evaluation of the risk of morphological changes. Our investigation showed that HPV16, 52 and 58 accounted for the top three genotypes resulting in cervical cytopathologic abnormalities, which was in accordance with data from other studies (Luo et al., 2020; Yan et al., 2021). Compared to the other genotypes, HPV16 was the major species in the ASC-H, HSIL and SCC cytology samples. Similar to other observations, HPV52 was the dominant genotype in cases with negative cytology and ASC-US (Li et al., 2020; Yan et al., 2021). Some HSIL were related to uncommon genotypes, such as HPV31 and HPV33. Approximately 16.67% of 126 patients with HPV33 had HSIL. In contrast, HSIL occurred in only 18 (approximately 9.52%) HPV18-positive cases. Colposcopy is recommended for women with HPV16/18, even if NILM is absent according to the American Society for Colposcopy and Cervical Pathology (ASCCP; Perkins et al., 2021). Based on our study, it is also appropriate to perform colposcopy screening in women with HPV52/58/31/33 in China.

The distribution of different pathological results for each genotype was similar to the distribution of cytopathological results. HPV16, 58, and 52 were also the major genotypes in women with histological abnormalities. Relative risk analysis demonstrated that women infected with HPV16, 31, 33, and 58 had an increased risk of HSIL compared with the other genotypes, which supports the above recommendation for additional colposcopy examinations in the population in China (Long et al., 2018; Yan et al., 2021). In our study, HPV16 and 18 were also the top two oncogenic genotypes associated with cervical cancer, consistent with the results of a previous study (Yan et al., 2021).

According to the guidelines from the American Cancer Society, HPV testing in combination with cytology or cytology alone should be phased out once full access to primary HPV examination for cervical cancer screening is available without barriers (Fontham et al., 2020). Therefore, HPV testing and prevention will play a more important role in epidemiological management in different regions. HPV16/18 were detected in 75.89% of cervical cancer samples in our study, suggesting that approximately 70% of cervical carcinomas could be prevented by bivalent or quadrivalent HPV vaccination (Gonzalez-Bosquet et al., 2020). Moreover, only 24 cases, accounting for 4.51% of cervical cancer patients, were found to be caused by high-risk genotypes not covered by the 9-valent vaccine. China and other 193 countries made the commitment to make sure that 90% of girls will be fully vaccinated by the age of 15 years in 2030 (Xia et al., 2020). However, the application of this vaccine is limited by low availability of imported vaccine and a long vaccination process (Yan et al., 2021). Although China has not integrated HPV vaccination into State immune programme, some provinces such as Fujian, Zhejiang and Guangdong have implemented free administration of domestic bivalent vaccine for girls under 15 years old since 2022. As a populous developing country, China’s national campaign on HPV vaccination is restricted by shortage of vaccine supply and high price of imported vaccine. Therefore, the development of sufficient and inexpensive domestic preventive HPV vaccines covering HPV16, 18, 31, 33, 39, 51, 52, 53, and 58 should be accelerated to achieved the goal.

The major limitation of our study was the fact that participants were enrolled from a single institution and not by randomized sampling of the population; therefore, the results might not be representative of all women in southern Hunan Province. In addition, some HPV-positive patients were lost to follow-up (e.g., attended other hospitals for morphological examination) and thus were not included in the morphological study, which resulted in a selection bias.

## Conclusion

This study analysed the prevalence of HPV and the age-specific HPV genotype distribution during 2017–2020 in southern Hunan Province. Moreover, the relationships between HPV genotypes and cervical morphological lesions were also analysed. Overall, HPV prevalence was 14.59% showing a decreased trend compared to previous study in the region. Bimodal distribution of age-specific HPV infection rate demonstrated females aged ≤20 or >60 years were susceptible to HPV. Significantly, HPV 52, 16 and 58 were main genotypes occurred in cervical abnormalities. Additionally, Infection of HPV 31, 33, 39, 51, and 53 should not be ignored in the follow-up management. Our study provides epidemiological data for the development of domestic HPV vaccines and future national campaign on HPV vaccination.

## Data availability statement

The raw data supporting the conclusions of this article will be made available by the authors, without undue reservation.

## Ethics statement

The studies involving human participants were reviewed and approved by the Ethics Committee of the First Affiliated Hospital of University of South China. Written informed consent from the participants’ legal guardian/next of kin was not required to participate in this study in accordance with the national legislation and the institutional requirements.

## Author contributions

ZL designed the study and drafted the manuscript. HL performed the HPV analyses. JZ, RH, and QZ collected and reviewed the data. FY performed the statistical analysis and reviewed the manuscript. All authors contributed to the article and approved the submitted version.

## Funding

This work was supported by The First Affiliated Hospital, Hengyang Medical School, University of South China.

## Conflict of interest

The authors declare that the research was conducted in the absence of any commercial or financial relationships that could be construed as a potential conflict of interest.

## Publisher’s note

All claims expressed in this article are solely those of the authors and do not necessarily represent those of their affiliated organizations, or those of the publisher, the editors and the reviewers. Any product that may be evaluated in this article, or claim that may be made by its manufacturer, is not guaranteed or endorsed by the publisher.

## Abbreviations

HPV: human papillomavirus
LBC: liquid-based cytology
LBP: liquid-based preparation
NILM: intraepithelial lesion or malignancy
ASC-US: atypical squamous cells of undetermined significance
ASC-H: atypical squamous cells, not excluding high-grade squamous intraepithelial lesion
AGC-NOS: atypical glandular cell-not otherwise specified
AGC-FN: atypical glandular cell-favour neoplasia
AC: adenocarcinoma
LSIL: low-grade squamous intraepithelial lesion
HSIL: high-grade squamous intraepithelial lesion
CIN: cervical intraepithelial neoplasia
CI: confidence interval
WHO: World Health Organization
